# Treatment outcomes of reduced-dose intravitreal ganciclovir for cytomegalovirus retinitis

**DOI:** 10.1186/s12879-016-1490-6

**Published:** 2016-04-18

**Authors:** Pitipol Choopong, Kamolporn Vivittaworn, Duanphen Konlakij, Somanus Thoongsuwan, Auengporn Pituksung, Nattaporn Tesavibul

**Affiliations:** Department of Ophthalmology, Faculty of Medicine Siriraj Hospital, Mahidol University, Bangkok, 10700 Thailand

**Keywords:** Cytomegalovirus retinitis, Ganciclovir, Intravitreal injections, Treatment outcomes

## Abstract

**Background:**

Cytomegalovirus retinitis (CMVR) is one of the most common opportunistic infection in immunocompromised individuals. Intravitreal ganciclovir injection has been used successfully but no standard regimen was established. Risks of drug toxicity, endophthalmitis, and injection-related complications increased with number and frequency of injection. The aim of this study is to evaluate the outcomes of reduced-dose intravitreal ganciclovir (2 mg/0.04 mL) for the treatment of CMVR.

**Methods:**

A prospective observational cohort study involving 67 eyes of 49 patients with CMVR was performed. Induction therapy involved intravenous ganciclovir (10 mg/kg/day) for 2 weeks unless contraindicated or patients refused. Patients were then treated with reduced-dose intravitreal ganciclovir every week for 4 weeks, and then every other week until the lesion healed. The patients’ demographic data were recorded, and vision parameters were examined every visit.

**Results:**

Twenty eyes (29.9 %) presented with initial visual acuities less than 6/60. The majority of patients were diagnosed with CMVR in zones 1 or 2 (63 eyes, 94 %), and, at least, one quadrant of the retina was involved (56 eyes, 83.6 %). Forty-one eyes (61.2 %) completely resolved after treatment within the 6-month follow-up. There was no significant difference in healing time, whether or not patients received induction treatment with intravenous ganciclovir (111.00 ± 12.96 vs 105.00 ± 28.32 days, *p* = 0.8). Five eyes (12.2 %) of patients with healed CMVR had visual acuities less than 6/60.

**Conclusions:**

Reduced-dose intravitreal ganciclovir is a safe and effective treatment option. It provides comparable results to other weekly regimens. Induction with intravenous ganciclovir is not crucial in a resolution of retinitis, although it may be necessary to reduce systemic cytomegalovirus loads and mortality rates.

**Trial registration:**

The trial was registered with Thai Clinical Trials Registry (TCTR) on 16 March 2016 – TCTR20160316001.

## Background

Cytomegalovirus (CMV) is a DNA virus of the Herpesviridae family. After primary infection, the virus becomes latent in monocytes and endothelial cells for the remaining lifetime of the patient. Reactivation of CMV infection may occur when the host immune system is compromised. The most common presentation is CMV retinitis (CMVR), especially in HIV/AIDS, lymphoma, and organ-transplanted patients, and primary immunodeficiency diseases. The most common manifestation of CMVR is the classic or fulminant form, described as segmental necrotic retinitis with intraretinal haemorrhage along the major vessels of the retina [1]. The two other manifestations, granular and perivascular forms, are less common. If left untreated, retinitis will progress, leading to blindness from the optic nerve or foveal involvement, and retinal detachment.

Ganciclovir is recommended for treatment of CMVR [2]. It interferes with DNA polymerase, resulting in inhibition of viral replication. The Food and Drug Administration of the US has approved two routes of administration of ganciclovir for treatment of CMVR, an intravenous route and an intraocular implant. The intravenous ganciclovir (IVG) showed a benefit of systemic CMV control and reduction of mortality rate but the major complication is agranulocytosis or pancytopenia. Intraocular implants demonstrated local control of CMVR in the affected eye for 6 months without systemic side effects [3, 4]. The need for retinal surgery and the high risk of retinal detachment make this method unfavourable. Both approved methods are expensive and may not be appropriate for low- or middle-income countries. Alternatively, intravitreal injection of ganciclovir (IVTG) has been introduced [5–7]. This method is easy and safe and provides fast and high intraocular drug concentrations. The reported dose varies from 0.2 to 5 mg of ganciclovir [5–11]. To date, there was no standard regimen in the treatment of CMVR with intravitreal ganciclovir injection. The recommended injection interval is twice weekly for 2 weeks, maintained with continuous weekly injections until the patient’s immune system reaches a reconstitution state and the lesion becomes a scar. The potential complications from intraocular injection include endophthalmitis, vitreous haemorrhage, and retinal vascular occlusion; additionally, retinal detachment increases with increased numbers and frequencies of injection [12].

Therefore, a less frequent injection should decrease the risk of these complications and also lessened the burden on compliance of patients and workload of healthcare personals, especially in low socioeconomic settings. In 2005, Yutthitham and Ruamviboonsuk reported a successful result of high-dose (4 mg/0.1 ml), alternate-week intravitreal injection of ganciclovir [11]. However, high dose (4 mg) ganciclovir could result in toxicity, for example, we reported a case of crystallization of 4-mg intravitreal ganciclovir injection leading to retinal arterial occlusion and optic atrophy [13]. With a consideration of toxicity from high dose ganciclovir, we consequently reduced the regimen to 2-mg ganciclovir. From our observation, CMVR lesion stopped in 2-4 weeks after induction with systemic ganciclovir. Therefore, in our regimen, the standard induction with intravenous ganciclovir was introduced for 2 weeks if the patient agreed to stay at the hospital and there was no contraindication. After systemic induction, 4 weekly injections were given before reduction to alternated week injection. We considered this regimen to balance the efficacy, side effects, and burden to patients and health workers in low-middle income settings as our institute. We thus proposed a reduced dose of four weekly 2-mg ganciclovir intravitreal injections, followed by alternate week maintenance for the treatment of CMVR, to reduce the frequency and side effects of the intraocular injection.

## Methods

This prospective observational cohort study was conducted at the CMVR clinic of Siriraj Hospital, from November 2009 to September 2012. The study was approved by the institutional review board of Siriraj Hospital, and adhered to the tenets of the Declaration of Helsinki. Patients over 18 years of age with CMVR were recruited. Exclusion criteria included previously treated CMVR or other concurrent retinal diseases. The diagnosis of cytomegalovirus retinitis (CMVR) was made clinically by indirect ophthalmoscopic findings. The CMVR findings included areas of necrotic retinal infiltration with or without haemorrhage. In addition, aqueous polymerase chain reaction (PCR) for herpes CMV was performed to confirm the diagnosis in some cases with controversial findings. After informed consent was obtained, the patient was encouraged to receive induction treatment in the hospital with IVG at 5 mg/kg/dose every 12 h for 2 weeks, except for patients who were contraindicated or were unable to remain at the hospital. After the induction, patients were treated with reduced-dose IVTG as directed. The reduced-dose IVTG regimen included weekly injections of 2 mg/0.04 mL ganciclovir for 4 weeks, followed by the same dosage every other week until there was complete resolution of CMVR.

The IVTG was prepared at the date of injection using the following protocol under sterile conditions. Ganciclovir (Cymevene®; 500 mg; Roche, Basel, Switzerland) was diluted with 10 mL of distilled water to give a 50-mg/mL solution. Ganciclovir (2 mg/0.04 mL) was drawn and kept in a 1-mL syringe. The injection was performed in the treatment room. After being anaesthetized with topical 0.5 % tetracaine eye drops, the patient’s eye was cleaned with 10 % povidone-iodine and applied with 5 % povidone-iodine eye drops before the operation. The intravitreal injection was performed 4 mm from the limbus, using a 30-gauge needle under sterile conditions. Topical 0.3 % ofloxacin eye drops were given immediately after the injection, four times daily for 3 days.

The patients’ demographic data were recorded, including age, sex, laterality, underlying diseases, HIV infection, CD4 count, and antiretroviral treatment. Best corrected Snellen visual acuity (VA), intraocular pressure (IOP) measurement, and a full eye examination using a slit-lamp biomicroscope and indirect ophthalmoscope were performed initially and at every follow-up visit. Full fundus photography was done initially and at 4-week intervals.

Statistical analysis was performed using descriptive statistics for the proportion of eyes with the resolution of CMVR within 6 months. A Kaplan–Meier graph and Cox regression analysis were used to evaluate median time-to-resolution and factors affecting healing times of CMVR. Subgroup analysis was compared between patients who received IVG with IVTG and with IVTG alone. Chi-squared and paired *t*-tests were used to compare the results between each group. All analyses were performed using SPSS software, version 18.0 (SPSS Inc., Chicago, IL, USA).

## Results

Sixty new CMVR cases were diagnosed from November 2009 to September 2012. Eleven patients were excluded because of concomitant retinal detachment, because they were already receiving standard IVG, or because of an inability to follow the proposed IVTG regimen. Sixty-seven eyes of 49 patients were analysed in the cohort. Twenty-six patients (53.1 %) received complete treatment with IVG followed by IVTG; the remaining patients received only IVTG. Twelve patients withdrew from the protocol (eight lost in follow-up, one because of low platelets, one with no light perception, and two died from sepsis). The patient demographic data are summarized in Table 1. The mean age was 39.5 ± 9.4 years, with a slight male preponderance (57.1 %). Thirty-one patients (63.3 %) presented with unilateral CMVR. Most of the unilateral cases (61.3 %) received IVTG without IVG. Forty-six patients (93.9 %) were HIV positive with median CD4 counts of 21 cells/mm^3^ (range, 1–482). Five HIV positive patients (10.9 %) had CD4 counts greater than 100 cells/mm^3^. Thirty-four patients (73.9 %) received antiretroviral treatment before the diagnosis of CMVR. Of 37 patients treated in Siriraj hospital, all patients received a fixed-dose combination of lamivudine (3TC) 150 mg, stavudine (d4T) 30 mg, and nevirapine (NVP) 200 mg before or concurrent with CMVR treatment. The three non-HIV cases included two patients with systemic lymphoma and one patient who had received a kidney transplant. Eight cases underwent aqueous aspiration for Herpes virus PCR. All of them were CMV positive. One patient had co-infection with VZV.

**Table 1:** Baseline characteristics of patients with cytomegalovirus retinitis

	IVTG alone	IVG + IVTG	Total
***Patient-specific characteristics***	***N = 23***	***N = 26***	***N = 49***
Age in years (mean ± SD)	40.52 ± 11.62	38.62 ± 7.01	39.51 ± 9.40
Male (%)	12 (52.17)	16 (61.54)	28 (57.14)
Bilateral involvement (%)	4 (17.39)	14 (53.85)	18 (36.73)
HIV infection (%)	20 (86.96)	26 (100)	46 (93.88)
Concurrent anti-retroviral therapy (%)	13 (56.52)	21 (80.77)	34 (69.39)
Mean initial CD4 (cells/mm^3^)(range)	49.50 (1–482)	45.60 (2–200)	47.23 (1–482)
***Eye-specific characteristics***	***N = 27***	***N = 40***	***N = 67***
Initial visual acuity (%)			
-6/60 or better	19 (70.37)	28 (70.00)	47 (70.15)
-worse than 6/60 to HM	6 (22.22)	10 (25.00)	16 (23.88)
-worse than HM	2 (7.41)	2 (5.00)	4 (5.97)
Initial IOP in mmHg (mean ± SD)	12.00 ± 5.90	10.58 ± 2.81	11.15 ± 4.34
Initial anterior chamber cell^a^ (%)			
-2+ or less	23 (85.19)	33 (82.50)	56 (83.58)
-more than 2+	4 (14.81)	7 (17.50)	11 (16.42)
Initial vitreous cell^a^ (%)			
-2+ or less	25 (92.59)	37 (92.50)	62 (92.54)
-more than 2+	2 (7.41)	3 (7.50)	5 (7.46)
Worst location involved^b^ (%)			
-Zone 1	12 (44.45)	21 (52.50)	33 (49.25)
-Zone 2	14 (51.85)	16 (40.00)	30 (44.78)
-Zone 3	1 (3.70)	3 (7.50)	4 (5.97)
Location size (%)			
-less than 25 %	6 (22.22)	5 (12.50)	11 (16.42)
-25–49 %	18 (66.67)	31 (72.50)	49 (73.13)
-50 % and more	3 (11.11)	4 (10.00)	7 (10.45)
Frosted branch angiitis presented (%)	17 (62.96)	30 (75.00)	47 (70.15)
Papillitis presented (%)	8 (29.63)	6 (15.00)	14 (20.89)
Foveal involvement (%)	9 (33.33)	13 (32.50)	22 (32.84)

*IVTG* intravitreal ganciclovir, *IVG* intravenous ganciclovir, *IOP* intraocular pressure, *HM* hand motion, *SD* standard deviation

^a^Anterior and vitreous reaction according to the Standardization of Uveitis Nomenclature guideline

^b^Worst zone of retina involved; Zone 1, lesions located in the area of the retina within 1500 microns from the edge of the optic nerve or within 3000 microns from the centre of the fovea. Zone 2, lesion located from the edge of zone 1 anteriorly to the circle of the vortex vein. Zone 3, lesion located anteriorly from zone 2 to the ora serrata

Forty-seven eyes (70.2 %) initially had a VA equal to or better than 6/60, while 20 eyes (29.9 %) presented with less than 6/60. The mean IOP before treatment was 11.15 ± 4.34 mmHg. Only one patient presented with IOP of more than 20 mmHg and was successfully treated with antiglaucoma eye drops. Most cases showed zero to moderate anterior and vitreous inflammation. There were 56 (83.58 %) and 62 (92.54 %) eyes with grade 2+ cells or less in the anterior and vitreous chambers, respectively.

Half of the patients were diagnosed with CMVR in zone 1 (33 eyes, 49.3 %). Forty-one eyes (61.2 %) had retinitis extending equally to or less than one quadrant. Forty-seven eyes (70.1 %) showed vasculitis before treatment in at least one of the four major vessels. Papillitis and foveal involvement were found in 14 eyes (20.9 %) and 22 eyes (32.8 %), respectively.

Complete resolution of CMVR was observed in 41 eyes (61.2 %) within a 6-month follow-up (Table 2). Excluding patients lost to follow-up, the resolution percentage was 73.2 %. The mean number of injections was 8.5 ± 3.1 times. The average time-to-resolution of CMVR was 97.2 ± 42.5 days (range, 21–188 days). In subgroup analysis, IVG with IVTG required slightly longer time to resolve than IVTG alone, although there was no statistically significant difference in time-to-resolution between the two groups (mean ± SD; 99.5 ± 45.3 vs 93.1 ± 38.3 days, respectively, *p* = 0.65). No recurrence was seen during the follow-up period. In cases of unilateral CMVR, there were no patients with a second eye involvement during the follow-up period. Thirty-four eyes (82.9 %) involving successful resolution of CMVR had a VA that was stable or improved after treatment. Thirty-six eyes (87.8 %) had a VA equal to or better than 6/60. The mean IOP was 11.6 ± 2.5 mmHg in eyes with complete resolution. During treatment, there were no patients with IOPs that exceeded 20 mmHg. In subgroup analyses, there were no statistical differences between patients treated with or without IVG, including the healing percentage, final VA, and final IOP (Table 2). Thirteen of 67 eyes experienced complications during treatment. Patients with CMVR resolution had fewer side effects (5/41 eyes, 12.2 %; one patient with transient acute visual loss, two patients with subconjunctival haemorrhages, and two patients with retinal detachments) than patients who failed the regimen (8/26 eyes, 30.8 %; one patient with permanent visual loss, two patients with severe pain, two patients with subconjunctival haemorrhages, one patient with retinal detachment, and two patients with vitreous haemorrhages).

**Table 2:** Results of reduced-dose intravitreal ganciclovir treatment for CMVR

	IVTG alone	IVG + IVTG	Total
Healed CMVR (%)	15/27 (55.56)	26/40 (65.00)	41/67 (61.19)
Visual acuity in healed CMVR (%)			
-6/60 or better	14 (93.33)	22 (84.62)	36 (87.81)
-worse than 6/60 to HM	1 (6.67)	3 (11.54)	4 (9.75)
-worse than HM	0 (0.00)	1 (3.84)	1 (2.44)
IOP in mmHg (mean ± SD)	12.20 ± 1.69	11.23 ± 2.86	11.59 ± 2.52
Complications (%)	2 (13.3)	3 (11.5)	5 (12.2)

*IVTG* intravitreal ganciclovir, *IVG* intravenous ganciclovir, *CMVR* cytomegalovirus retinitis, *HM* hand motion, *IOP* intraocular pressure, *SD* standard deviation

**Fig. 1:**
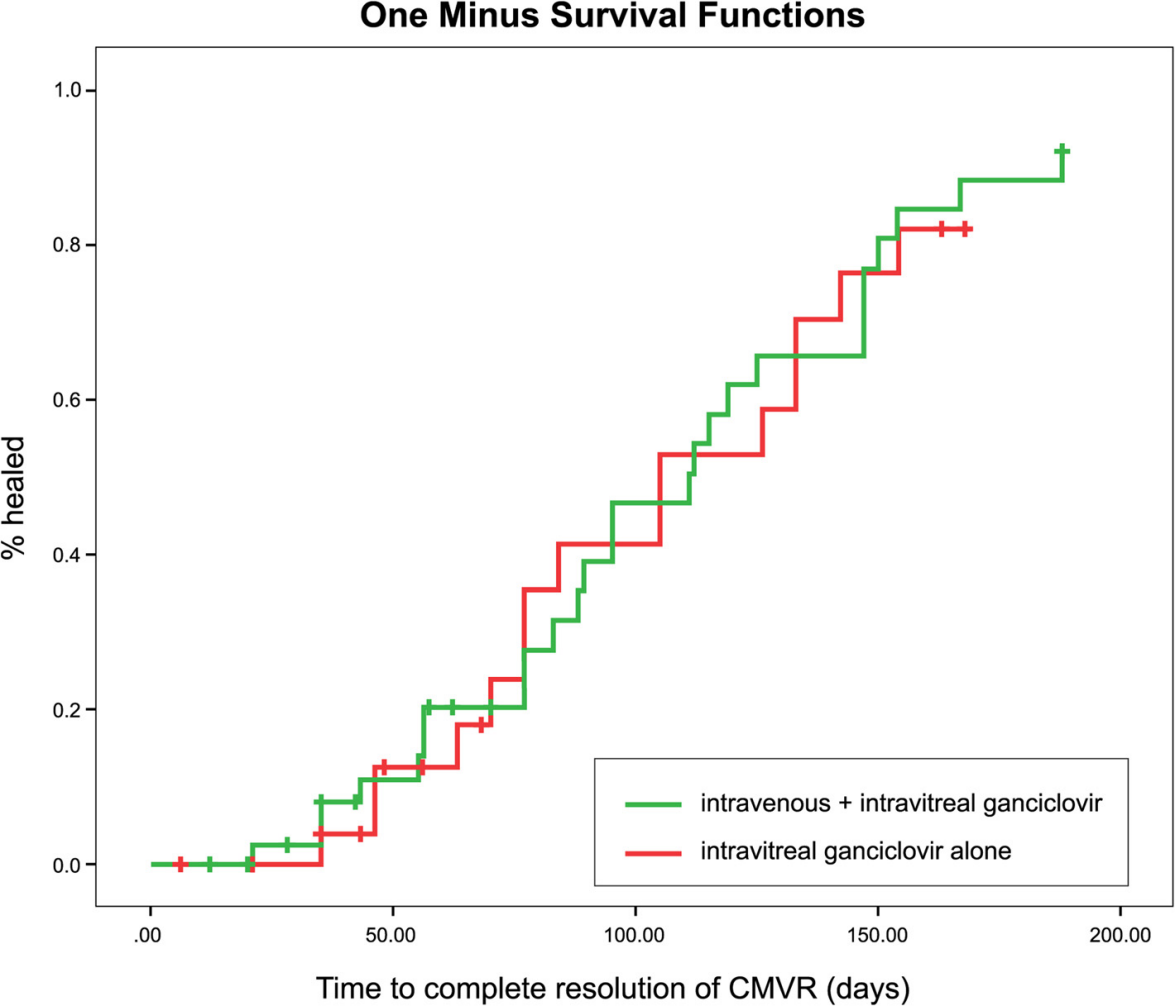
Kaplan–Meier graph showing complete resolution of cytomegalovirus (CMVR) lesions in eyes that received reduced-dose intravitreal ganciclovir injection alone, compared with eyes that received reduced-dose intravitreal combined with intravenous ganciclovir injection

Kaplan–Meier survival analysis showed that the regimen reached a median resolution time at 111.00 ± 12.82 days (95 % CI; 85.88–136.12). There was no significant difference in resolution time whether or not the patient received IVG induction treatment (*p* = 0.8, log-rank test) (Fig. 1). Multivariate Cox regression analysis indicated a shorter resolution time in patients initially presenting with an age younger than 45 years (HR 0.40; 95 % CI 0.17–0.93, *p* = 0.03), with no vasculitis (HR 3.45; 95 % CI 1.52–7.83, *p* = 0.003), or with no foveal involvement (HR 2.65; 95 % CI 1.14–6.11, *p* = 0.023) (Table 3). The VA stabilization or improvement was affected by the absence of vasculitis (HR 2.55; 95%CI 1.30–5.00, *p* = 0.007) and by the absence of retinitis in zone 1 (HR 0.51; 95 % CI 0.28–0.94, *p* = 0.03) (Table 4).

**Table 3:** Univariate and multivariate Cox regression analyses of factors involving healing time

Factors	Univariate		Multivariate	
	*p*	*HR (95 % CI)*	*p*	*HR (95 % CI)*
Age younger than 45 years old	0.013	2.86 (1.25–6.67)	0.033	2.5 (1.08–5.88)
Male	0.247	1.46 (0.77–2.67)	0.382	1.51 (0.60–3.83)
Induction with intravenous ganciclovir	0.798	1.09 (0.57–2.07)	0.494	1.26 (0.65–2.44)
Initial CD4 > 100	0.287	1.58 (0.68–3.67)		
Concurrent anti-retroviral therapy	0.324	1.43 (0.70–2.92)		
Initial visual acuity of 6/60 or better	0.335	1.42 (0.69–2.92)		
No zone 1 involvement	0.717	1.12 (0.60–2.08)	0.091	0.50 (0.23–1.12)
Area of retinitis less than 25 %	0.258	1.45 (0.76–2.78)	0.500	1.41 (0.52–3.83)
No foveal involvement	0.162	1.64 (0.82–3.29)	0.023	2.65 (1.14–6.11)
No disc involvement	0.345	1.43 (0.68–3.01)		
Frosted branch angiitis not presented	0.007	2.58 (1.29–5.16)	0.003	3.45 (1.52–7.83)

*HR* hazard ratio, *CI* confidence interval

^a^Zone 1, lesions located in the area of the retina within 1500 microns from the edge of the optic nerve or within 3000 microns from the centre of the fovea

**Table 4:** Univariate and multivariate Cox regression analyses of factors involving vision stabilization or improvement

Factors	Univariate		Multivariate	
	*p*	*HR (95 % CI)*	*p*	*HR (95 % CI)*
Age younger than 45 years old	0.054	2.04 (0.99–4.17)	0.062	1.96 (0.96–4.00)
Male	0.044	1.87 (1.02–3.42)	0.197	1.54 (0.80–2.95)
Induction with intravenous ganciclovir	0.372	0.77 (0.44–1.36)	0.533	0.83 (0.46–1.49)
Initial CD4 > 100	0.467	0.68 (0.24–1.92)		
Concurrent anti-retroviral therapy	0.958	1.02 (0.55–1.88)		
Initial visual acuity of 6/60 or better	0.312	0.74 (0.41–1.33)		
No zone 1 involvement^a^	0.122	0.64 (0.36–1.13)	0.030	1.96 (1.07–3.58)
Area of retinitis less than 25 %	0.252	0.71 (0.39–1.28)	0.192	0.59 (0.27–1.30)
No foveal involvement	0.885	0.96 (0.53–1.72)		
No disc involvement	0.670	1.15 (0.60–2.22)		
Frosted branch angiitis not presented	0.017	2.18 (1.15–4.13)	0.007	2.55(1.30–5.00)

*HR* hazard ratio, *CI* confidence interval

^a^Zone 1, lesions located in the area of the retina within 1500 microns from the edge of the optic nerve or within 3000 microns from the centre of the fovea

## Discussion

Based on the present study, a reduced dose regimen for IVTG, with a low percentage of complications, was effective in controlling CMVR. Although systemic anti-CMV therapy is the major treatment for CMVR, it is not usually available in middle- and low-income countries such as Thailand. IVG and oral valganciclovir are very expensive for the general population, and these treatments have limited use because of systemic side effects. Alternative treatments with various doses of IVTG have been successful in many countries [5–11, 14–16], but the suggested protocols still need frequent follow-up visits. It is difficult for patients with low socioeconomic status to follow these protocols, because of other burdens, especially transportation costs.

Our reduced-dose protocol attempted to decrease expenses, patient visits, and medical personnel workloads while producing reasonable results. It demonstrated 73.2 % complete resolution of CMVR, without recurrence, within 6 months of treatment. The average healing time was about 14 weeks (3–27 weeks) with an average of 8.3 injections. Resolution of the lesion needed longer times than weekly maintenance protocols. This agrees with other reports of alternate week regimens [11, 15]. This is probably due to insufficient drug concentration in the vitreous that is below the 50 % minimal inhibitory concentration (MIC_50_) during the second week, although other factors may help cessation of CMVR, such as antiretroviral treatment and improvement of the patient’s immune status. Increasing the dosage of ganciclovir may elevate the vitreous concentration and shorten the healing time, but may also result in optic nerve or macular toxicity [13, 17]. Using subgroup analyses, there was no statistical significance in healing times and healing percentages between eyes with or without IVG induction.

At the conclusion of treatment, VA was stable or improved in 73 % of all eyes. In patients with complete resolution, VA improved or stabilized in 83 % of eyes. Eyes treated with IVTG alone resulted in even better vision preservation than those with IVG induction. This is probably because of the smaller area of retinitis. Our protocol showed better results in preserving vision than previous reports of IVG treatment [11, 15, 16, 18]. A previous report, involving alternate week injections of 4 mg/0.1 mL, showed a better resolution percentage of 82.4 % within about 12 weeks (2–36 weeks) but only 51 % of patients had vision preserved [11]. It demonstrated that a higher dose of ganciclovir injection may increase the percentages of resolution, but also increases the risk of ganciclovir toxicity, with a decreased final vision.

Young et al. reported a 100 % success percentage with three biweekly 2 mg/0.1 mL of IVTG followed by weekly IVTG maintenance. However, it resulted in a 7 % relapse with median relapse time of 42 weeks [19]. In our cohort, there was no relapse after stopping the medication, but the follow-up time was limited to 24 weeks, which was probably insufficient to evaluate the actual relapse time of IVTG. Nonetheless, it was much longer than the 8-week relapsing period of the IVG. Owing to cost limitations, we did not evaluate the CD4 counts at the time of CMVR resolution, but a cessation of injections without recurrence or progression of lesions may signify the recovery of the patient’s immune system.

The complication percentage from our cohort was 16.4 % compared with 8.3 to 20.6 % using other IVTG regimens [11, 15, 16, 19]. Most complications were minor, including subconjunctival haemorrhage, pain, and transient visual loss, except for three eyes with rhegmatogenous retinal detachment and one eye with an acute permanent visual loss. There was no endophthalmitis in our study while it was reported in other IVTG studies with comparable patient numbers [11, 16]. The absence of endophthalmitis in our study may result from improvements in injection techniques and use of antibiotics.

In our study, the median time-to-resolution of CMVR was about 16 weeks. There was no significant difference whether or not patients received IVG prior to IVTG. Therefore, our findings indicated that IVG induction was not needed for the resolution of CMVR. However, because IVG helps in eradication of the systemic CMV viral load, prevents the risk of extraocular or second eye involvement, and reduces the mortality rate of patients, we still encourage induction with IVG if available or not contraindicated [20].

Based upon regression analyses, patients younger than 45 years old, without vasculitis, and without foveal lesions were associated with shorter time-to-resolution of CMVR. Gender, VA, induction with IVG, CD4 counts, and extent or zone of retinitis at presentation showed no associations with healing times. Younger patients may possess a better immune function, despite their CD4 counts, and may, therefore, respond faster to treatment. Vascular and foveal involvement may indicate more complicated cases, requiring longer healing periods. Patients with retinitis not involving zone 1 and with an absence of vasculitis were associated with improvement or preservation of the final VA. These results indicate that the central retina and vascular structure play crucial roles in visual prognosis.

There were limitations in our study, including it being a nonrandomized trial with a small sample size. However, we selectively excluded CMVR patients with other retinal diseases to minimize the bias of the treatment results. Our study is the first prospective cohort study from Thailand; we thus believe our results could be relevant to real ophthalmic clinical settings specifically for low- to middle-income countries. A larger randomised control trial should be conducted in the future to provide a better understanding of the different regimens.

## Conclusions

In conclusion, our reduced-dose regimen of IVTG for CMVR represents an efficacious and safe alternative therapy, especially for resource-scarce settings. The resolution of CMVR was achieved in favourable numbers of patients within 6 months, When compared with other regimens, the final visual outcome was better, while the complication rates were similar. IVG induction may be required to eradicate systemic CMV viral loads, but is not necessary for treatment of ocular CMVR. With an average of eight injections per person, both workloads and patient visits are reduced. We, therefore, recommend this reduced-dose regimen for treatment of CMVR, particularly in low- or middle-socioeconomic settings.

## Availability of data and materials

The data analysed in this study can be accessed by sending a request to the corresponding author.

## Ethics approval

Siriraj Hospital Institutional Review Board, Siriraj hospital Mahidol University, Bangkok, Thailand #Si 158/2010. Consent form was obtained from each patient.

## Abbreviations

CMV: Cytomagalovirus
CMVR: Cytomegalovirus retinitis
IOP: Intraocular pressure
IVG: Intravenous ganciclovir
IVTG: Intravitreal ganciclovir
VA: Visual acuity

## References

[1] Moorthy RS. 2011-2012 Basic and Clinical Science Course, Section 9: Intraocular Inflammation and Uveitis (Basic & Clinical Science Course). Revised edition. San Francisco, CA: American Academy of Ophthalmology; 2011. p.204

[2] Whitley RJ, Jacobson MA, Friedberg DN, Holland GN, Jabs DA, Dieterich DT (1998). Guidelines for the treatment of cytomegalovirus diseases in patients with AIDS in the era of potent antiretroviral therapy: recommendations of an international panel. International AIDS Society-USA. Arch Intern Med.

[3] Anand R, Nightingale SD, Fish RH, Smith TJ, Ashton P (1993). Control of cytomegalovirus retinitis using sustained release of intraocular ganciclovir. Arch Ophthalmol.

[4] Martin DF, Parks DJ, Mellow SD, Ferris FL, Walton RC, Remaley NA (1994). Treatment of cytomegalovirus retinitis with an intraocular sustained-release ganciclovir implant. A randomized controlled clinical trial. Arch Ophthalmol.

[5] Henry K, Cantrill H, Fletcher C, Chinnock BJ, Balfour HH (1987). Use of intravitreal ganciclovir (dihydroxy propoxymethyl guanine) for cytomegalovirus retinitis in a patient with AIDS. Am J Ophthalmol.

[6] Daikos GL, Pulido J, Kathpalia SB, Jackson GG (1988). Intravenous and intraocular ganciclovir for CMV retinitis in patients with AIDS or chemotherapeutic immunosuppression. Br J Ophthalmol.

[7] Cochereau-Massin I, LeHoang P, Lautier-Frau M, Zazoun L, Marcel P, Robinet M (1991). Efficacy and tolerance of intravitreal ganciclovir in cytomegalovirus retinitis in acquired immune deficiency syndrome. Ophthalmology.

[8] Young SH, Morlet N, Heery S, Hollows FC, Coroneo MT (1992). High dose intravitreal ganciclovir in the treatment of cytomegalovirus retinitis. Med J Aust.

[9] Hodge WG, Lalonde RG, Sampalis J, Deschênes J (1996). Once-weekly intraocular injections of ganciclovir for maintenance therapy of cytomegalovirus retinitis: clinical and ocular outcome. J Infect Dis.

[10] Arevalo JF, Garcia RA, Mendoza AJ (2005). High-dose (5000-microg) intravitreal ganciclovir combined with highly active antiretroviral therapy for cytomegalovirus retinitis in HIV-infected patients in Venezuela. Eur J Ophthalmol.

[11] Yutthitham K, Ruamviboonsuk P (2005). The high-dose, alternate-week intravitreal ganciclovir injections for cytomegalovirus retinitis in acquired immune deficiency syndrome patients on highly active antiretroviral therapy. J Med Assoc Thai.

[12] Sampat KM, Garg SJ (2010). Complications of intravitreal injections. Curr Opin Ophthalmol.

[13] Choopong P, Tesavibul N, Rodanant N (2010). Crystallization after intravitreal ganciclovir injection. Clin Ophthalmol.

[14] Visser L (2003). Managing CMV, retinitis in the developing world. Community Eye Health.

[15] Teoh SC, Ou X, Lim TH (2011). Intravitreal Ganciclovir Maintenance Injection for Cytomegalovirus Retinitis: Efficacy of a Low-Volume, Intermediate-Dose Regimen. Ophthalmology.

[16] Ausayakhun S, Yuvaves P, Ngamtiphakom S, Prasitsilp J (2005). Treatment of cytomegalovirus retinitis in AIDS patients with intravitreal ganciclovir. J Med Assoc Thai.

[17] Saran BR, Maguire AM (1994). Retinal toxicity of high dose intravitreal ganciclovir. Retina.

[18] Montero MC, Pastor M, Buenestado C, Lluch A, Atienza M (1996). Intravitreal ganciclovir for cytomegalovirus retinitis in patients with AIDS. Ann Pharmacother.

[19] Young S, Morlet N, Besen G, Wiley CA, Jones P, Gold J (1998). High-dose (2000-μg) intravitreous ganciclovir in the treatment of cytomegalovirus retinitis. Ophthalmology.

[20] Kempen JH, Jabs DA, Wilson LA, Dunn JP, West SK, Tonascia J (2003). Mortality risk for patients with cytomegalovirus retinitis and acquired immune deficiency syndrome. Clin Infect Dis.

